# Indoor resting behavior of *Aedes aegypti* (Diptera: Culicidae) in northeastern Thailand

**DOI:** 10.1186/s13071-023-05746-9

**Published:** 2023-04-14

**Authors:** Chadapond Seang-arwut, Yupa Hanboonsong, Vithee Muenworn, Joacim Rocklöv, Ubydul Haque, Tipaya Ekalaksananan, Richard E. Paul, Hans J. Overgaard

**Affiliations:** 1 Department of Entomology, Faculty of Agriculture, Khon Kaen University, Khon Kaen, Thailand; 2 Heidelberg Institute of Global Health & Heidelberg Interdisciplinary Centre for Scientific Computing, Heidelberg University, Heidelberg, Germany; 3 Department of Public Health and Clinical Medicine, Section of Sustainable Health, Umeå University, Umea, Sweden; 4 Rutgers Global Health Institute, New Brunswick, NJ USA; 5 Department of Biostatistics and Epidemiology, School of Public Health, Rutgers University, Piscataway, NJ USA; 6 Department of Microbiology, Faculty of Medicine, Khon Kaen University, Khon Kaen, Thailand; 7 UMR2000, Ecology and Emergence of Arthropod-Borne Pathogens Unit, Institut Pasteur, Université Paris Cité, CNRS, 75015 Paris, France; 8 Faculty of Science and Technology, Norwegian University of Life Sciences, Ås, Norway

**Keywords:** Mosquito abundance, Vector control, Indoor residual spraying, Height, Room, Gecko

## Abstract

**Background:**

*Aedes aegypti* is a vector of several arboviruses, notably dengue virus (DENV), which causes dengue fever and is often found resting indoors. *Culex* spp. are largely nuisance mosquitoes but can include species that are vectors of zoonotic pathogens. Vector control is currently the main method to control dengue outbreaks. Indoor residual spraying can be part of an effective vector control strategy but requires an understanding of the resting behavior. Here we focus on the indoor-resting behavior of *Ae. aegypti* and *Culex* spp. in northeastern Thailand.

**Methods:**

Mosquitoes were collected in 240 houses in rural and urban settings from May to August 2019 at two collection times (morning/afternoon), in four room types (bedroom, bathroom, living room and kitchen) in each house and at three wall heights (< 0.75 m, 0.75–1.5 m, > 1.5 m) using a battery-driven aspirator and sticky traps. Household characteristics were ascertained. Mosquitoes were identified as *Ae. aegypti*, *Aedes albopictus* and *Culex* spp. Dengue virus was detected in *Ae. aegypti*. Association analyses between urban/rural and within-house location (wall height, room), household variables, geckos and mosquito abundance were performed.

**Results:**

A total of 2874 mosquitoes were collected using aspirators and 1830 using sticky traps. *Aedes aegypti* and *Culex* spp. accounted for 44.78% and 53.17% of the specimens, respectively. Only 2.05% were *Ae. albopictus*. *Aedes aegypti* and *Culex* spp. rested most abundantly at intermediate and low heights in bedrooms or bathrooms (96.6% and 85.2% for each taxon of the total, respectively). Clothes hanging at intermediate heights were associated with higher mean numbers of *Ae. aegypti* in rural settings (0.81 [SEM: 0.08] vs. low: 0.61 [0.08] and high: 0.32 [0.09]). Use of larval control was associated with lower numbers of *Ae. aegypti* (yes: 0.61 [0.08]; no: 0.70 [0.07]). All DENV-positive *Ae. aegypti* (1.7%, 5 of 422) were collected in the rural areas and included specimens with single, double and even triple serotype infections.

**Conclusions:**

Knowledge of the indoor resting behavior of adult mosquitoes and associated environmental factors can guide the choice of the most appropriate and effective vector control method. Our work suggests that vector control using targeted indoor residual spraying and/or potentially spatial repellents focusing on walls at heights lower than 1.5 m in bedrooms and bathrooms could be part of an integrated effective strategy for dengue vector control.

**Graphical Abstract:**

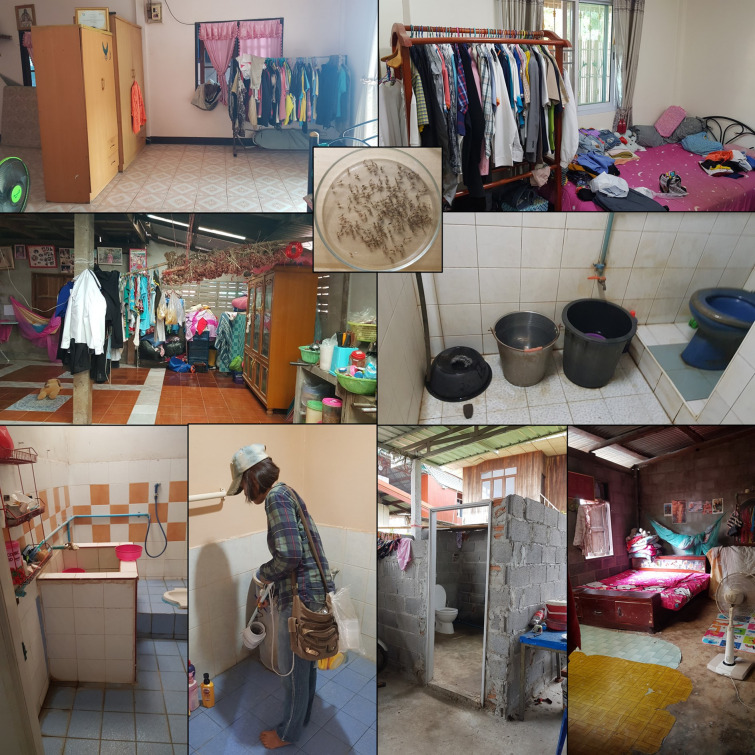

**Supplementary Information:**

The online version contains supplementary material available at 10.1186/s13071-023-05746-9.

## Background

Dengue is the most widespread mosquito-borne viral disease in the world. The number of dengue cases reported to the World Health Organization has increased more than eight-fold over the last two decades [1]. An estimated 50 million dengue infections occur annually, and approximately 2.5 billion people live in dengue endemic countries [2]. *Aedes aegypti* (Linnaeus, 1762) is a tropical and subtropical mosquito species widely distributed globally. It is a primary vector of the dengue virus (DENV) and is well adapted to completing its entire life cycle within urban areas and around houses, primarily feeding on humans. It also transmits yellow fever, Zika and chikungunya viruses. *Aedes albopictus* is a secondary vector of DENV and, although more rural, also exhibits peridomestic resting and biting behaviors.

The mainstay of dengue vector control interventions in endemic settings, including Thailand, focuses on the immature stages, supplemented with space-spraying insecticides to target the adult mosquito population during an outbreak. Although space-spraying does reduce mosquito numbers, there are no studies showing that such methods are effective in reducing the number of cases [3–5]. Indoor residual spraying (IRS) is a method commonly used in malaria vector control, providing a long-term means of impacting mosquito populations and thus more logistically and economically viable than intermittent fogging; it has also recently become of interest in the dengue control community [6]. The effectiveness of IRS relies on knowledge of where mosquitoes rest, and therefore targeted IRS should focus on areas where adult mosquitoes are most likely to rest. Adult *Ae. aegypti* generally rest indoors rather than outdoors [7, 8], especially on the lower parts of walls, and can vary depending on the type of room (kitchen vs. bedroom for example) [9, 10]. Resting preference is believed to be influenced by the type of surface, with a preference for cloth, wood and cement [7]. However, house structures differ worldwide, and global generalizations may not be applicable. As far as we know, there are currently no reports on the resting behaviors of adult *Ae. aegypti* in Southeast Asia. Therefore, the main objective of this study was to determine the most preferred indoor resting locations of *Ae. aegypti* in urban and rural houses and associated environmental factors to provide information that can be used for effective vector control and dengue prevention. In addition to *Ae. aegypti*, we also analyzed the numbers of *Culex* spp. collected because of their relatively high number and importance, particularly as nuisance species, even though several species can also be vectors of important pathogens. We also compared two mosquito collection methods to assess the relative effectiveness of aspiration versus sticky traps. Adult mosquito collections are generally performed by aspiration methods, but sticky traps offer an alternative, passive low-labor method, and although subjected to a limited number of studies, have been used in conjunction with the gravid *Aedes* trap [11–13]. Finally, as there are few studies exploring the effect of geckos as a mosquito control tool in the field [14], we took advantage of the fact that sticky traps also collected geckos and assessed the relationship between sticky-trap-collected geckos and mosquito abundance.

## Methods

### Study area

**Fig. 1:**
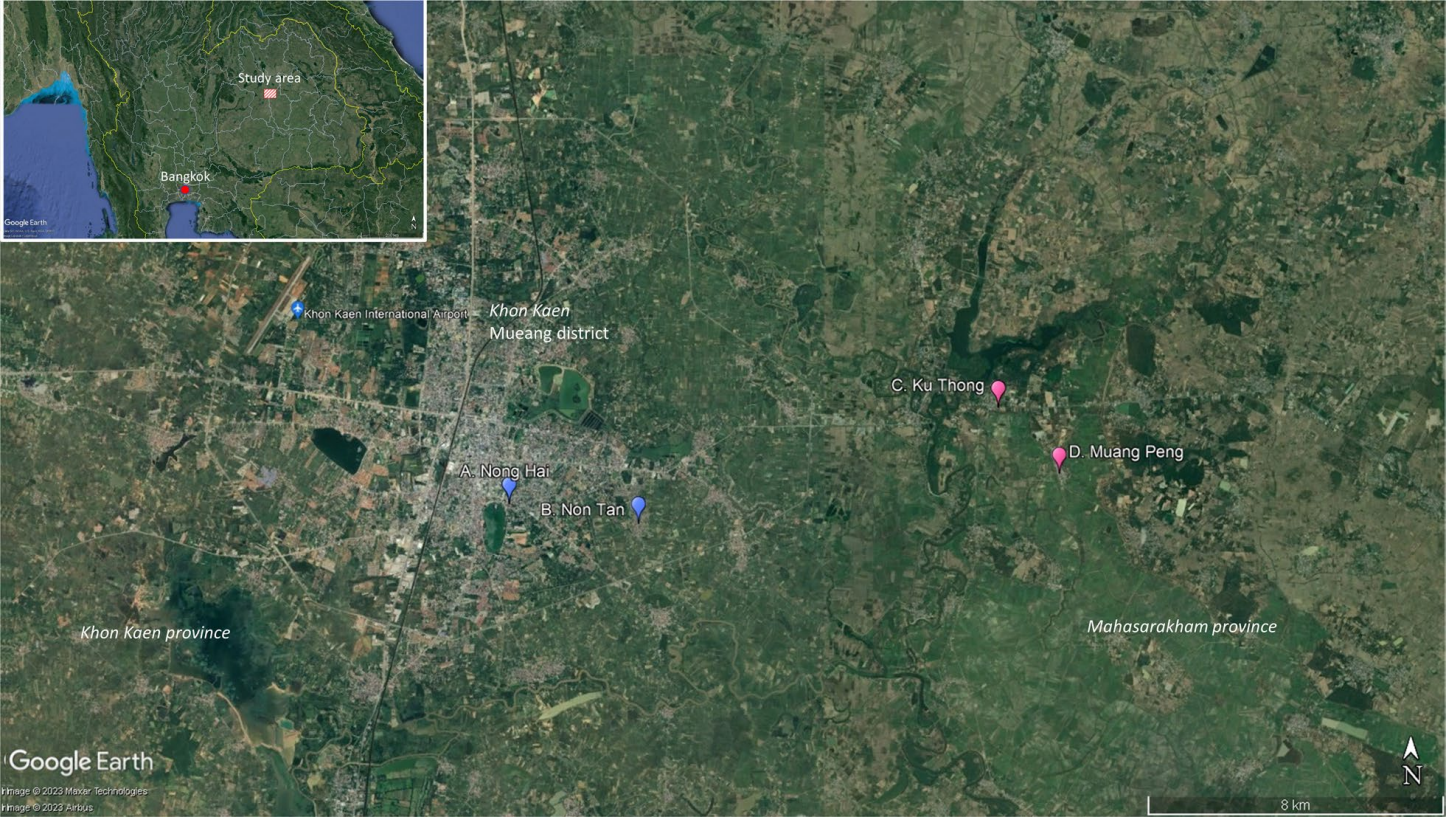
Study sites in urban areas (blue markers: Khon Kaen Mueang district, Khon Kaen province) and rural areas (purple markers: Chiang Yuen district, Mahasarakham province). **A** Nong Hai village, **B** Non Tan village, **C** Ku Thong village, **D** Muang Peng village

The study was carried out in northeastern Thailand in two rural sites, Ku Thong village (16.4421N, 102.9652E) and Muang Peng village (16.4261N, 102.9806E) in Chiang Yuen district, Mahasarakham province, and in two urban sites, Nong Hai (16.4141N, 102.8744E) and Non Tan (16.4189N, 102.8418E) in Mueang district, Khon Kaen province (Fig. 1). The study areas are dengue-endemic, with typical seasonal increases during the rainy season and occasional outbreaks [15]. The average minimum and maximum seasonal temperatures are 16.7 °C (December–January) and 36.4 °C (April–May). The monthly minimum and maximum rainfall vary from 0 mm (dry season: November–April) to 240 mm (wet season: May–October). This study was conducted during the rainy season. During the study period, there was no instance of public health intervention through mosquito control in response to any occurring dengue outbreak.

### Study design and data collection

Cross-sectional entomological surveys using mechanical aspirators were carried out in 60 randomly selected households (from household village rosters) in each of the four sites, i.e., 120 in the urban area and 120 in the rural area, totaling 240 households. Collections were done sequentially during the rainy season in May–August 2019 in the following order: May, rural Ku Thong (Fig. 1C); June, urban Nong Hai (Fig. 1A); July, rural Muang Peng (Fig. 1D); and August, urban Non Tan (Fig. 1B). Collections were completed in 18–20 days at each study site. In addition, five randomly selected houses per village (a total of 20 households) were used for collecting mosquitoes using sheets with insect glue (see below).

Mosquitoes were collected in the morning between 08:00 and 12:00 in 30 of the selected houses in each site and in 30 different houses in the afternoon between 13:00 and 17:00 using a mechanical battery-driven aspirator (as described in [9]). Because of refusal for afternoon collection in some sites (both rural villages), collection ultimately yielded 37 houses with morning samples and only 23 houses with afternoon samples in Ku Thong and 29 houses in the afternoon and 31 in the morning in Muang Peng. Thirty different houses were sampled in the morning and in the afternoon for both urban sites. Mosquitoes were collected on the lower part (0–0.75 m) of the walls for 10 min in each of the bedroom, bathroom, kitchen and living room, respectively. This procedure was then repeated on the middle part of the wall (0.75–1.5 m) in each room, followed by the upper parts (> 1.5 m) of the walls in each room. The upper parts were reached by fixing the aspirator to a long handle. The total aspiration time per room was 30 min.

**Fig. 2:**
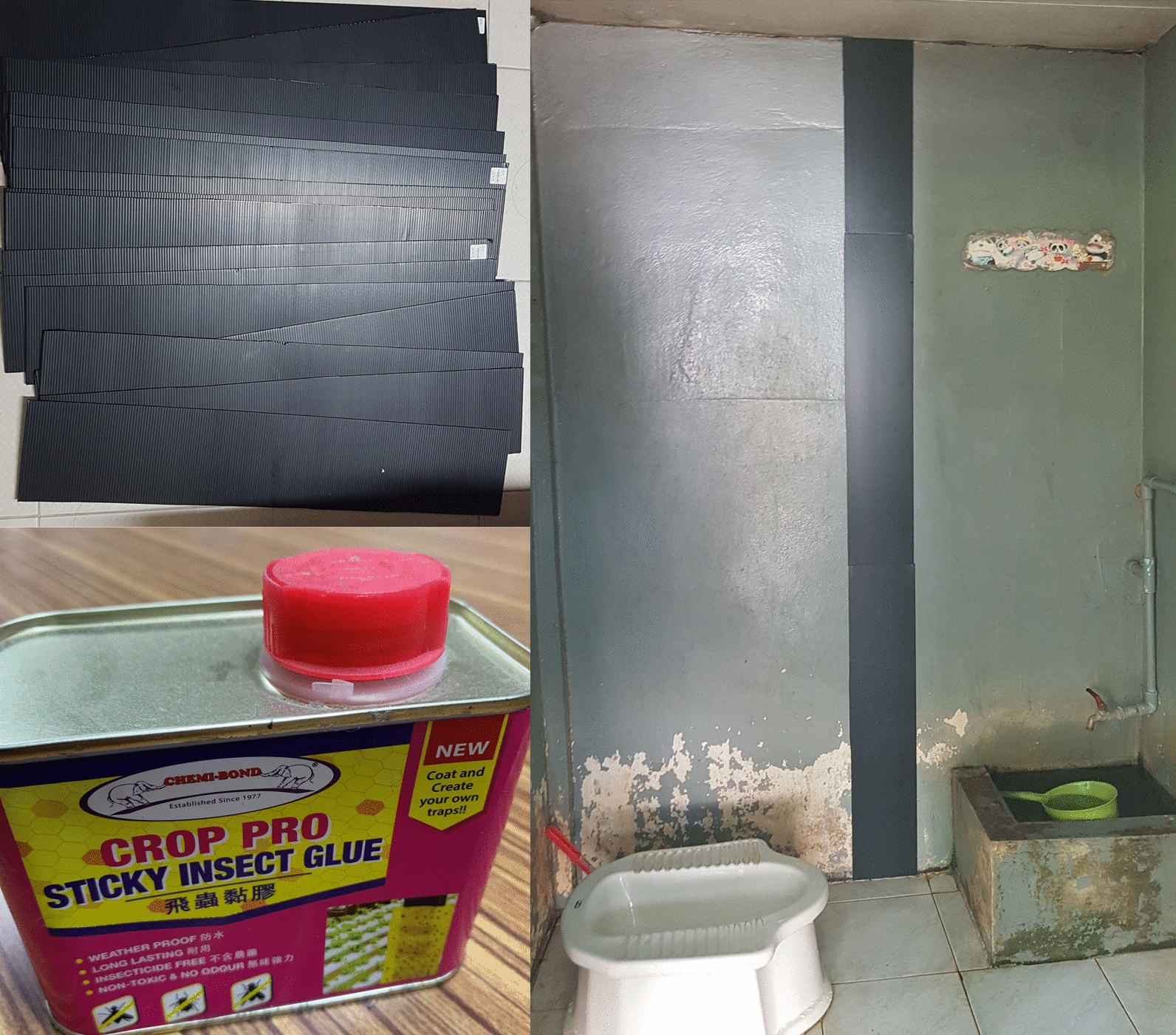
The sticky glue trap. Upper left: 1-m-long black corrugated plastic sheets. Right: sticky trap placed on a toilet wall. Lower left: insect glue

Sticky traps were deployed in five randomly selected houses in each of the two rural and two urban sites. The sticky traps were set up 1 week after these houses had been aspirated. Sticky traps consisted of ~ 10-cm-wide strips of black polypropylene corrugated sheets, covered with 10 clear plastic sheets (0.1 × 0.3 m) spread with Crop Pro Sticky Insect Glue (Chemibond Enterprise Sdn Bhd, Malaysia) and placed vertically, extending from the floor to a height of 3 m (or lower if obstructed by the ceiling), on a suitable wall area unobstructed by furniture or clothes in the bedroom, bathroom, living room and kitchen (one trap/room) (Fig. 2). The sticky trap sheets were replaced every week during the same period as the aspirations took place for a total collection time of 3 weeks. Sticky traps were transported to the laboratory for species identification. In addition to mosquitoes, sticky traps caught house geckos, the numbers of which were recorded. Geckos were not identified to species, but the most common species in residential areas in Southeast Asia include the common Asian house gecko *Hemidactylus frenatus* and the flat-tailed house gecko *Hemidactylus platyurus* [16].

### Mosquito processing

After collection, mosquitoes were placed in collection cups reinforced by adhesive tape to avoid any possible escape. Each cup was labeled with household identification (ID), room type, height, time and date. Cups were stored in a Styrofoam box and transported back to the laboratory where they were killed at −20 °C, identified and sexed and blood-fed status determined (blood-fed or non-blood-fed) under a stereomicroscope. Species identification was made by morphological characteristics using keys by Rueda [17]. Mosquitoes on sticky traps were counted and identified to species using a magnifying lens. All specimens were identified as *Ae. aegypti*, *Ae. albopictus* or *Culex* spp., except those that could not be identified due to their state.

### Detection of DENV in mosquitoes

DENV identification in a total of 405 female blood-fed *Ae. aegypti*, collected by aspiration from both the rural and urban study areas, was conducted by reverse transcription quantitative polymerase chain reaction (RT-qPCR) assay. An additional 17 blood-fed mosquitoes were recovered from the sticky traps and in a state to be processed (out of the 194 identified as blood-fed). The mosquito head/thorax was separated from the abdomen and kept for later analysis if needed. The abdomens of these mosquitoes were pooled with approximately 10 abdomens per pool based on the date of collection, yielding a total of 45 pools. Viral RNA (ribonucleic acid) in each pool was sequestered using a QIAamp Viral RNA Mini Kit (QIAGEN, Valencia, CA, USA). Complementary DNA (cDNA) (deoxyribonucleic acid) was synthesized from 1 µg of the isolated viral RNA template with a DENV consensus primer (D2) [18], according to the manufacturer’s protocol of the SuperScript™ III First-Strand Synthesis System (Invitrogen, Waltham, MA, USA). The qPCR reaction was performed with a total of 10 µl mixture, containing 1 × SsoAdvanced™ Universal SYBR^®^ Green Supermix (Bio-Rad, Hercules, CA, USA), 0.25 µM of each D1 and D2 primer, and 50 ng of cDNA template. The amplification cycle was executed with 35 cycles of 95 °C for 15 s, 55 °C for 30 s and 72 °C for 45 s on the C1000 Touch™ Thermal Cycler (Bio-Rad, Hercules, CA, USA). The head/thorax of individual mosquitoes in the DENV-positive pools were retested to determine whether they were positive, and subsequently subjected to viral serotype identification. As described by Lanciotti and colleagues [18], DENV serotypes 1–4 were detected by universal DENV primer (D1) and specific reverse primers, including TS1, TS2, TS3 and TS4, respectively. RNA from DENV-infected *Ae. aegypti* mosquitoes was used as a positive control, and distilled water was retrieved to control the contaminated reaction.

### Household characteristics

A household questionnaire was used to collect data on households and their members by interview of the household head. Questions included: how many people lived in the household, the occupation of the head of the household, number of livestock in/around the house (ducks, chickens, pigs, cows, buffalo), number of pet animals (dogs, cats), sources of drinking and non-drinking water (piped water, groundwater, rainwater, bottled water/drinking water vending machines, other), type of house (single house one floor, single house two floors, commercial building, townhouse, apartment, other), number of rooms, wall construction (plaster, cement/bricks, burned bricks, wood, other), house cleanliness (poor, intermediate, good), level of wind flow (high, intermediate, low/none), toilet type (bowl toilet, squat toilet, other) and toilet location (indoors, outdoors), presence of window screens, presence of open eave gaps, and larval and adult mosquito control activities and frequency. The degree of darkness was estimated as light, intermediate or dark in each room where mosquitoes were collected. Similarly, the quantity (none or little, intermediate, a lot) and height of hanging clothes were estimated by eye for each room where clothes were found hanging. House cleanliness, wind flow, darkness, amount of clothes were estimated subjectively by the first author.

### Data analysis

The association of area (urban vs. rural), village and resting location (room and height), and time (morning and afternoon) with male and female mosquito numbers was analyzed by fitting a generalized linear mixed (log-linear, i.e., Poisson distribution) model (GLMM) with house fitted as the random factor. A Wald statistic, which follows a *χ*^2^ distribution, was calculated. A comparison of levels within a factorial variable was performed through calculation of a *t* statistic (the difference between the mean divided by the standard error of the differences). A dispersion parameter was estimated in the model fit to account for any overdispersion of the data. To compare the efficiency of the two mosquito capture methods (aspiration vs. sticky traps) GLMM log-linear regression was fitted with house as the random factor and area (urban/rural), village, room, height and capture methodology as explanatory variables, and natural log (ln)-transformed collection duration (i.e., 30 min for aspirator and 30,240 min (3 × 7 days × 24 h × 60 min) for the sticky trap) as an offset. The association of socioenvironmental variables and number of mosquitoes collected by aspirator was analyzed by fitting a GLMM log-linear regression with house ID and room nested within house ID as random factors. The variables were first fitted in univariable analyses and then those found to be associated at *P* < 0.2 were fitted in a multivariable model. All such variables were fitted in the full model and then the non-significant variables were removed from the model in a stepwise manner until the final adequate model with only significant variables was obtained. Urban and rural areas were analyzed separately because of the very large differences in many of the variables across these two types of areas, notably animal husbandry. A Bonferroni-corrected *P*-value threshold for the multiple statistical tests (38 household variables) performed was calculated as *P* = 0.0013. The number of geckos caught on sticky traps was analyzed as an explanatory variable for association with the numbers of mosquitoes caught. Associations were first assessed by univariable fitting of the number of geckos with the total number of mosquitoes caught on sticky traps only, then with the aspirator mosquito catch number data from the same houses. Finally, we performed a multivariable association analysis of mosquito number, including gecko number and significant socioenvironmental variables. All analyses were performed in Genstat version 20 software [19].

## Results

### Collection by aspirator

A total of 2874 mosquitoes were collected using aspirators (Table 1). *Aedes aegypti* and *Culex* spp. accounted for the majority of mosquitoes, 44.78% and 53.17%, respectively, with *Ae. albopictus* contributing only 2.05% (59 mosquitoes, mainly from the rural sites). There were significantly more *Ae. aegypti* collected in the rural villages than the urban villages (959 vs. 328, *χ*^2^ = 61.8, *df* = 1, *P* < 0.001). In addition, the second urban village (Non Tan) had even lower abundance than the first urban village (Nong Hai) (105 mosquitoes vs. 223). More female than male *Ae. aegypti* were collected overall (708 vs. 579, *χ*^2^ = 7.99, *df* = 1, *P* = 0.005). The abundance of *Culex* spp. was also higher in rural villages (992 vs. 615, *χ*^2^ = 17.5, *df* = 1, *P* < 0.001), but there was significant variation between villages in the rural and urban sites (*χ*^2^ = 9.96, *df* = 2, *P* = 0.008).

**Table 1:** Number of mosquitoes (%) collected by mechanical battery-driven aspirator and sticky traps in rural and urban areas in northeastern Thailand in 2019

Species	Methods	Areas	No. of mosquitoes			
			Female	Male	Sum	Total
*Ae. aegypti*	Aspirator	Rural	524 (74.0)	435 (75.1)	959 (74.5)	
		Urban	184 (26.0)	144 (24.9)	328 (25.5)	
		Sum	708 (100.0)	579 (100.0)	1287 (100.0)	
	Sticky trap	Rural	334 (64.1)	280 (64.7)	614 (64.4)	
		Urban	187 (35.9)	153 (35.3)	340 (35.6)	
		Sum	521 (100.0)	433 (100.0)	954 (100.0)	2241
*Ae. albopictus*	Aspirator	Rural	36 (83.7)	12 (92.3)	51 (91.5)	
		Urban	7 (16.3)	1 (7.7)	8 (8.5)	
		Sum	43 (100.0)	13 (100.0)	59 (100.0)	
	Sticky trap	Rural	0	4 (100.0)	4 (100.0)	
		Urban	0	0	0	
		Sum	0	4 (100.0)	4 (100.0)	63
*Culex* spp.	Aspirator	Rural	515 (71.8)	414 (52.3)	929 (60.8)	
		Urban	202 (28.2)	378 (47.7)	599 (39.2)	
		Sum	717 (100.0)	792 (100.0)	1528 (100.0)	
	Sticky trap	Rural	293 (62.1)	262 (57.7)	555 (63.6)	
		Urban	179 (37.9)	192 (42.3)	317 (36.4)	
		Sum	472 (100.0)	454 (100.0)	872 (100.0)	2400
Overall	Aspirator	Sum	1468 (59.7)	1384 (60.8)	2874 (61.1)	
	Sticky trap	Sum	993 (40.3)	891 (39.2)	1830 (38.9)	
		Total	2461 (100.0)	2275 (100.0)	4704 (100.0)	4704

**Fig. 3:**
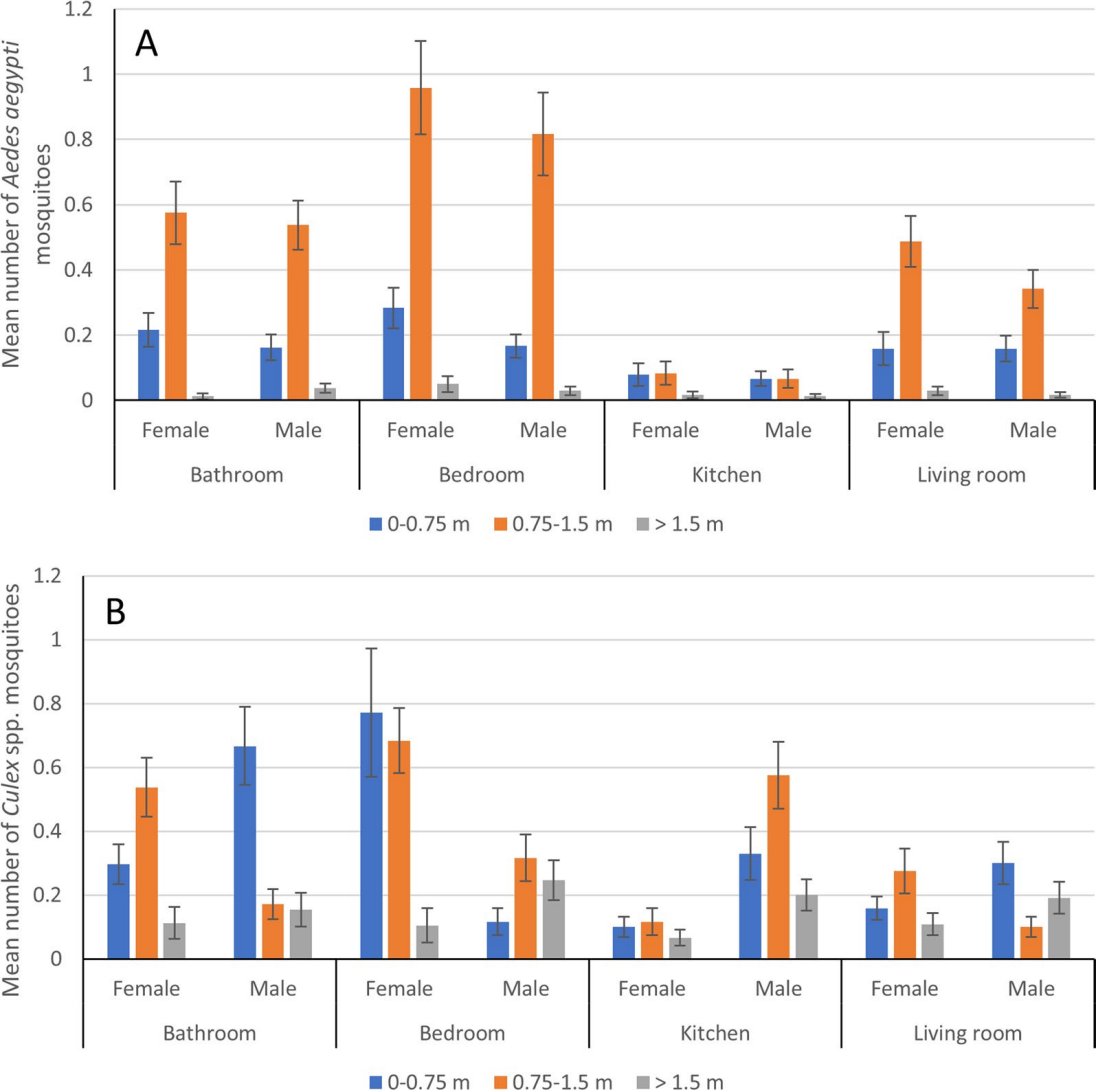
Mean number of female and male **A** *Ae. aegypti* and **B** *Culex* spp. mosquitoes aspirated at different heights in different rooms. Error bars show standard errors of the mean

Male and female *Ae. aegypti* were most abundant at intermediate heights in rooms where they were most prevalent (i.e., all rooms except the kitchen) (Fig. 3A). Overall, in decreasing order for abundance by height: 0.75–1.5 m > 0–0.75 m > 1.5 + m (all *t* statistics for the three among-height comparisons, *T* > 8.5, *P* < 0.001). In decreasing order of abundance were bedroom > bathroom = living room > kitchen (*t*-statistics for the five significant comparisons *T* > 3.1, *P* < 0.01). Both males and females showed the same distribution in heights and rooms.

*Culex* mosquitoes were more abundant at the two lower heights than at the higher height (*t* statistics for the two significant comparisons *T* > 5.8, *P* < 0.001). There was no difference in the number of male and female *Culex* mosquitoes caught (*χ*^2^ = 0.01, *df* = 1, *P* = 0.903). Although female *Culex* were found predominantly in the bedroom and bathroom, males showed very different room and height distributions (Fig. 3B). The full data set summaries of numbers of mosquitoes caught according to place, time and height for *Ae. aegypti* and *Culex* spp. are shown in Additional file 1: Table S1 and Additional file 2: Table S2.

There were very few *Ae. albopictus* mosquitoes overall, but there was a significant association with more *Ae. albopictus* present in the living room (total 15 specimens) and kitchen (nine specimens) than in the bedroom (six specimens) and bathroom (four specimens) (living room vs. bathroom *T* = 7.39, *P* < 0.001; living room vs. bedroom *T* = 5.99, *P* < 0.001; kitchen vs. bathroom *T* = 4.24, *P* < 0.001). There was no significant association with height (*χ*^2^ = 0.59, *P* = 0.745).

**Fig. 4:**
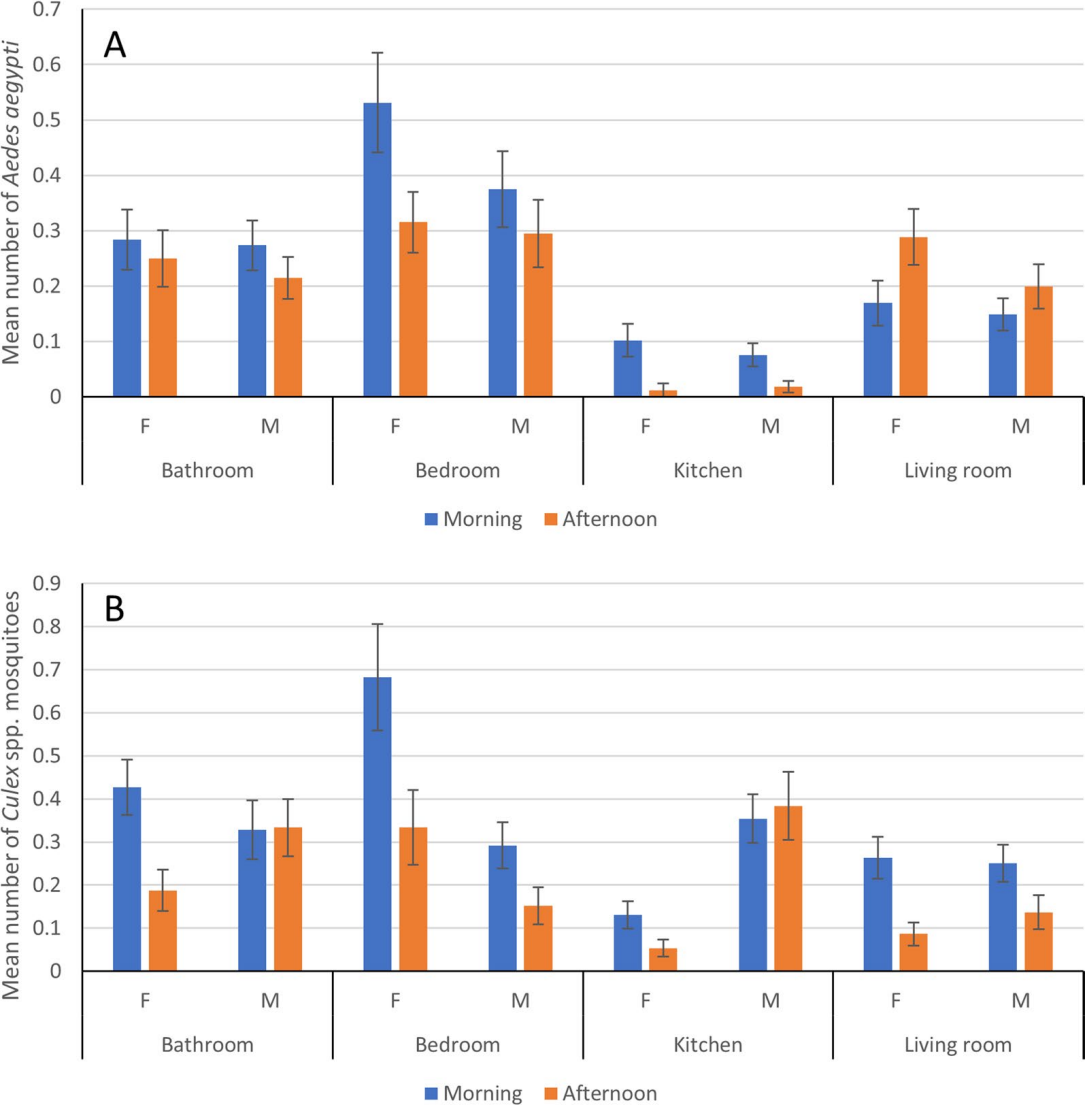
Mean number of female and male **A** *Ae. aegypti* and **B** *Culex* spp. mosquitoes aspirated in different rooms in the morning and the afternoon. Error bars show standard errors of the mean

The abundance of *Ae. aegypti* decreased from morning to afternoon in the bathroom, bedroom and kitchen but increased in the living room (Fig. 4A). These changes were significant for the female mosquitoes in the bedroom (*T* = 2.25, *P* < 0.05), the kitchen (*T* = 2.71, *P* < 0.01) and the living room (*T* = 2.42, *P* < 0.05). By contrast, the abundance of *Culex* spp. decreased from morning to afternoon in all rooms (*χ*^2^ = 15.24, *df* = 1, *P* < 0.001) (Fig. 4B). For *Ae. albopictus*, numbers decreased from morning to afternoon, but this was not significant (*χ*^2^ = 3.70, *df* = 1, *P* = 0.053).

**Fig. 5:**
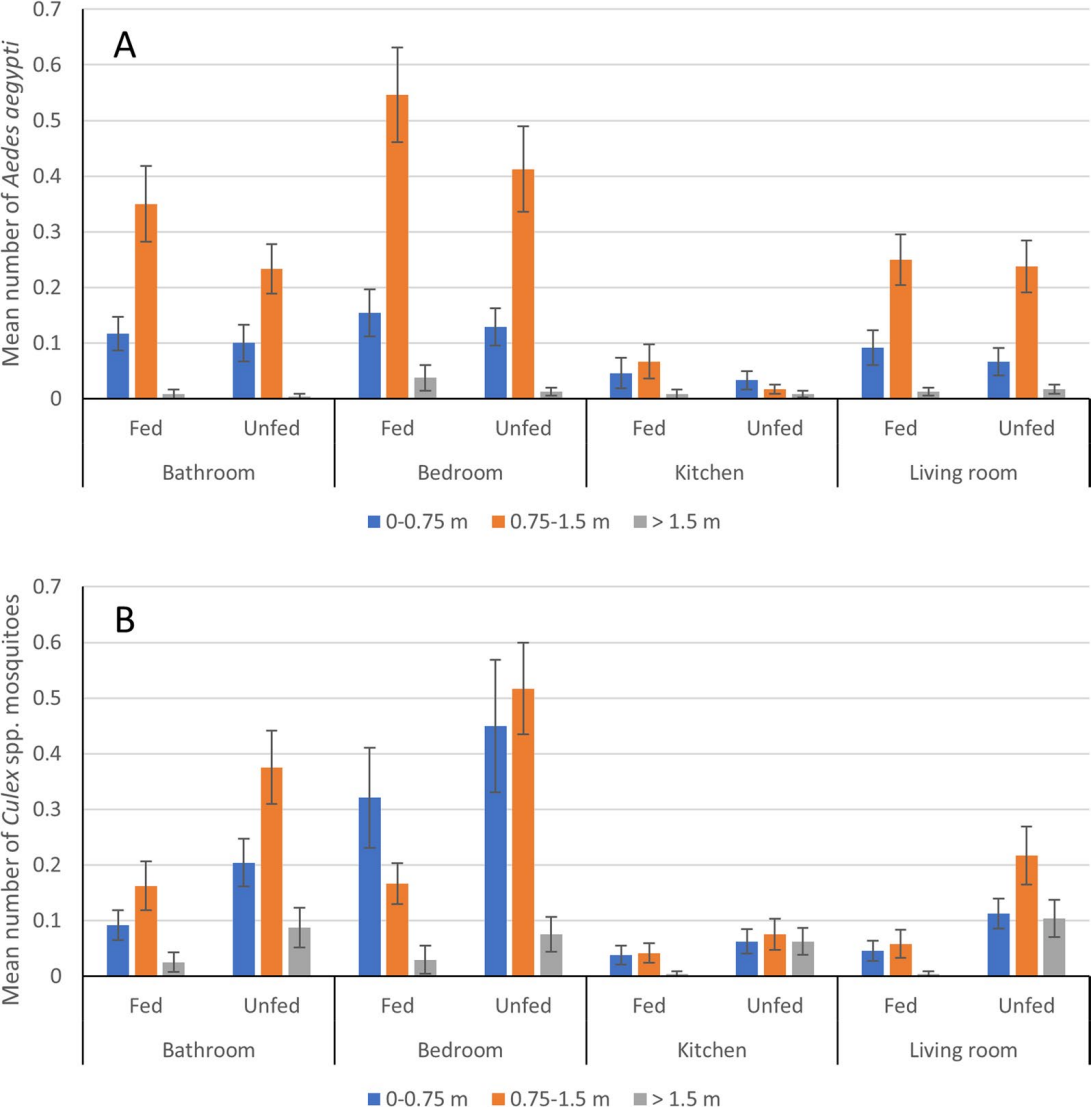
Mean number of fed and unfed **A** female *Ae. aegypti* and **B** female *Culex* spp. mosquitoes aspirated at different heights in different rooms. Error bars show standard errors of the mean

There were more blood-fed *Ae. aegypti* collected than unfed (*χ*^2^ = 9.35, *df* = 1, *P* = 0.002), but they were not differentially distributed by height (*χ*^2^ = 0.47, *df* = 2, *P* = 0.791) or room (*χ*^2^ = 2.75, *df* = 3, *P* = 0.432) (Fig. 5A). By contrast, fewer blood-fed *Culex* spp. female mosquitoes were collected than unfed (*χ*^2^ = 86.6, *df* = 1, *P* < 0.001) (Fig. 5B), there was a distinctly different height distribution for blood-fed mosquitoes in the bedroom compared to the other rooms. There were too few blood-fed *Ae. albopictus* for analysis.

### Sticky traps

A total of 1830 identifiable mosquitoes were collected by sticky traps (Table 1). *Aedes aegypti* and *Culex* spp. accounted for 52.1% and 47.7%, respectively, with *Ae. albopictus* contributing only 0.2% (only four mosquitoes, all from the urban sites). Less than 5% of the mosquitoes were unable to be identified. The full data set summaries of numbers of mosquitoes caught according to place, time and height for *Ae. aegypti* and *Culex* spp. are shown in Additional file 3: Table S3 and Additional file 4: Table S4.

**Fig. 6:**
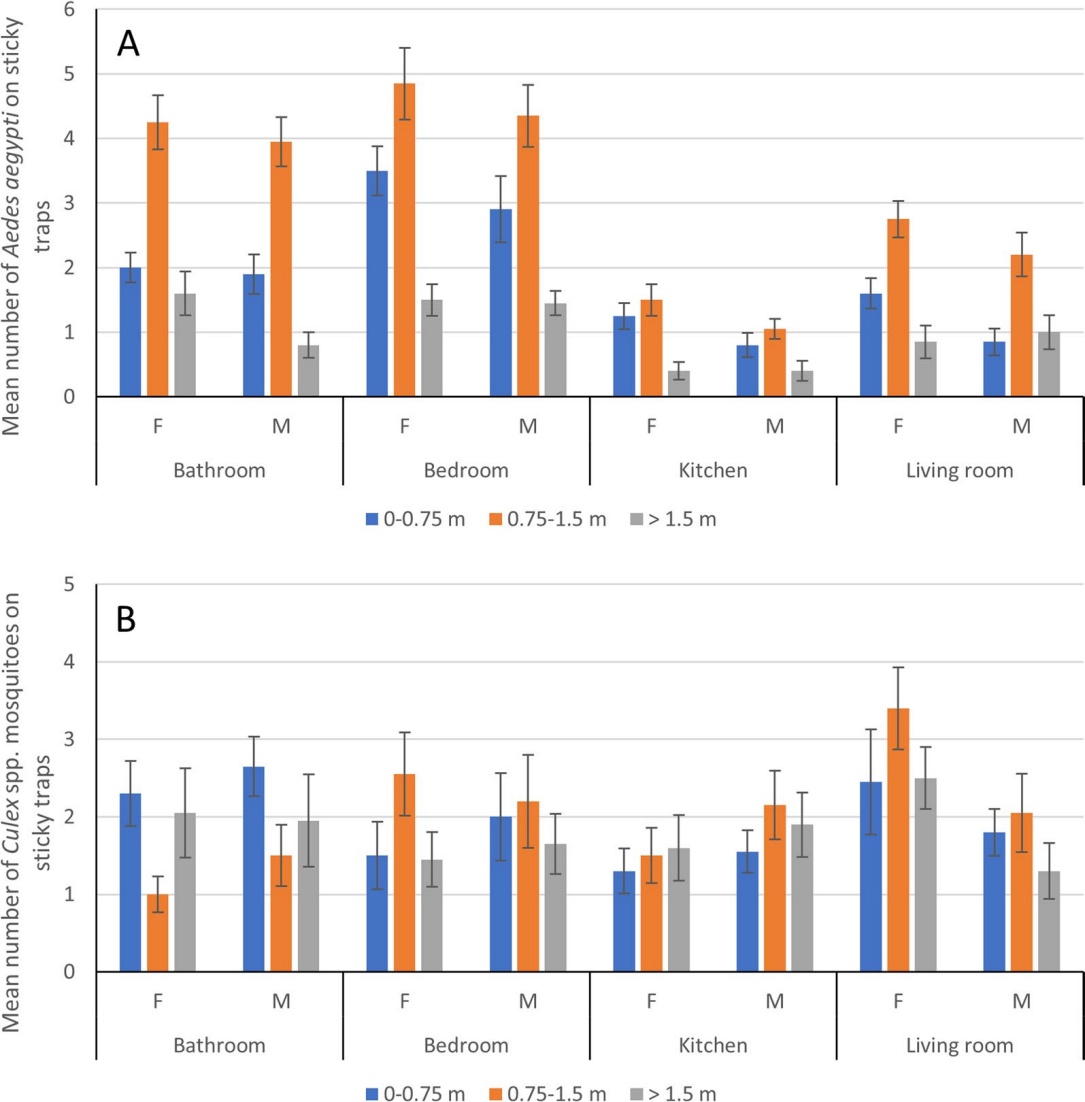
Mean number of female and male **A** *Ae. aegypti* and **B** *Culex* spp. mosquitoes on sticky traps at different heights in different rooms. Error bars show standard errors of the mean

*Aedes aegypti* abundance, as measured by sticky traps, was higher in the rural areas (*N* = 614) than in the urban areas (*N* = 340) (*χ*^2^ = 60.46, *df* = 1, *P* < 0.001), but did not differ further at the village level (*χ*^2^ = 0.92, *df* = 2, *P* = 0.63). There were more female than male mosquitoes (*χ*^2^ = 10.2, *df* = 1, *P* = 0.002), but no sex differences in height or room resting preference were found. There were significant differences in overall resting behavior, irrespective of sex, for both rooms (*χ*^2^ = 195.4, *df* = 3, *P* < 0.001) and heights (*χ*^2^ = 212.02, *df* = 2, *P* < 0.001) (Fig. 6A). The mosquito abundance in rooms and by height was in decreasing order: bedroom > bathroom > living room > kitchen (all *t* statistics *T* > 3.5, *P* < 0.001) and 0.75–1.5 m > 0–0.75 m > 1.5 + m (all *t* statistics *T* > 7.0, *P* < 0.001), respectively. There was no interaction between room and height for mosquito abundance.

*Culex* spp. sticky trap abundance was higher in the rural areas (*N* = 555 vs. *N* = 371) (*χ*^2^ = 18.06, *df* = 1, *P* < 0.001) but did not differ further at the village level (*χ*^2^ = 0.92, *df* = 2, *P* = 0.63). The numbers of female and male *Culex* mosquitoes did not significantly differ (*χ*^2^ = 0.17, *df* = 1, *P* = 0.68). There was a small interaction effect between room and height (*χ*^2^ = 14.16, *df* = 6, *P* = 0.03), reflecting the relatively lower mid-height abundance in the bathroom as compared to the other rooms (Fig. 6B). Otherwise, there were no significant differences in abundance by height (*χ*^2^ = 2.01, *df* = 2, *P* = 0.38) or room (*χ*^2^ = 5.27, *df* = 3, *P* = 0.154).

Only four *Ae. albopictus* were collected and thus were not analyzed.

### Comparison of mosquito collections by aspirator and sticky traps

Overall, in houses where mosquitoes were collected both by aspiration and sticky traps, the sticky traps collected more mosquitoes (*Ae. aegypti*: aspirator mean: 0.58, standard error of the mean [SEM] 0.13 vs. sticky trap mean: 3.98, SEM: 0.22. *Culex* spp. aspirator mean: 0.91, SEM: 0.19 vs. sticky trap mean: 3.86, SEM: 0.19). However, per sampling sticky traps were deployed for a lot longer (i.e., 3 times 7 days vs. 30 min). For the comparison, only aspirator mosquito collections from houses that had sticky traps deployed were used. Taking into account the differences between area, village, room and height, the aspirator method was more efficient per sampling time effort in collecting *Ae. aegypti* (*χ*^2^ = 1165.0, *df* = 1, *P* < 0.001) and *Culex* spp. (*χ*^2^ = 1372.6, *df* = 1, *P* < 0.001) mosquitoes. Aspirators collected a mean of 0.019 *Ae. aegypti/*min and 0.029 *Culex* spp.*/*min, whereas sticky traps collected a mean of 1.3 × 10^–4^ *Ae. aegypti*/min and 1.28 × 10^–4^ *Culex* spp./min. Although the efficiency of capture was higher at all heights, for *Ae. aegypti* there was a significant interaction effect where aspiration was even better at higher heights (vs. lowest height, *T* = 1.98, *P* = 0.048; vs. intermediate height, *T* = 3.38, *P* < 0.001).

### DENV-positive mosquitoes

Of the 422 *Ae. aegypti* females assessed for DENV infection, only five specimens (1.7%) were positive for the virus. This was too few to analyze with respect to their distribution in rooms and across heights. Two specimens were positive for DENV-1 (0.7%), one for DENV-3 (0.3%), and one with a mixed DENV-1 and DENV-3 infection (0.3%). One specimen was positive for DENV-1, DENV-2, and DENV-3 combined (0.3%). All DENV-positive mosquitoes were collected by aspiration in May 2019 in the rural village of Ku Thong, Mahasarakham province. These five specimens were found in one positive pool of 10 mosquitoes. Three positive mosquitoes were found in the living rooms of two houses, and two mosquitoes were found in the bedroom of one house. All five positive mosquitoes were found at a height of 0.75–1.5 m.

### Association of socioenvironmental variables

Summary household information collected using the questionnaire is shown in Additional file 5: Table S5. There were several notable differences between rural and urban areas. Overall, there were more people per household in urban settings (urban mean: 4.08 standard deviation [SD]: 1.71 vs. rural mean: 3.37, SD 1.58). Urban household heads were predominantly people with permanent jobs, shopkeepers and casual laborers (87%), whereas rural household heads were predominantly farmers and casual laborers. Almost no urban houses had any livestock, and relatively few rural houses had livestock (15%). Approximately half of the rural houses had wooden walls (53%), whereas 82% of urban houses had plaster walls. Eighty-seven percent of rural houses had squat toilets versus 67% of urban houses having bowl toilets. Eighty-seven percent of urban houses had window screens, whereas only 12% of rural houses had screens. Larval control either with temephos or via cleaning of containers was carried out more frequently (> 90% on a weekly basis) in urban areas than in rural areas (50% of houses at a monthly rate).

The *P*-values for the univariable analyses of the association of socioenvironmental variables with *Ae. aegypti* or *Culex* spp. mosquito numbers are shown in Additional file 6: Table S6 and Additional file 7: Table S7. In urban settings, in the final adequate multivariable model, only use of repellent (yes/no) was associated with total *Ae. aegypti* number, and the Yes category was found to be associated with increased numbers of mosquitoes (*χ*^2^ = 5.29, *df* = 1, *P* = 0.022). In rural settings, the following variables were found to be associated with higher *Ae. aegypti* number: use of temephos for larval control with low use (only every 3 months) was associated with higher numbers of mosquitoes (*χ*^2^ = 9.55, *df* = 3, *P* = 0.024); cement walls (*χ*^2^ = 8.62, *df* = 2, *P* = 0.014), outdoor toilets (*χ*^2^ = 5.75, *df* = 1, *P* = 0.017) and increasing number of rooms (*χ*^2^ = 8.91, *df* = 1, *P* = 0.003). Clothing location was also associated with mosquito numbers (*χ*^2^ = 12.78, *df* = 2, *P* = 0.002), with intermediate height of hung clothing being associated with more mosquitoes than lower or higher levels (intermediate vs. low levels: *T* = 2.19, *P* < 0.05; intermediate vs. high levels: *T* = 3.25, *P* < 0.01). With 38 variables analyzed and one multivariable analysis would yield a Bonferroni-corrected *P*-value of *P* = 0.0013. Thus, the associations observed above must be taken with caution.

For *Culex* spp. mosquitoes in urban settings, the following variables were found to be associated with higher mosquito numbers: absence of open eaves (*χ*^2^ = 6.34, *df* = 1, *P* = 0.012), infrequent use of fogging (every 3 months) (*χ*^2^ = 25.97, *df* = 2, *P* < 0.001) and low levels of wind flow (*χ*^2^ = 9.83, *df* = 2, *P* = 0.008). In rural settings, the use of fogging (*χ*^2^ = 16.9, *df* = 1, P < 0.001) was associated with lower numbers of mosquitoes, and the quantity of hung clothing (*χ*^2^ = 6.23, *df* = 2, *P* = 0.045; none/little vs. a lot, *T* = 2.46, P < 0.05) was associated with higher numbers of mosquitoes. Shopkeeping as the household occupation was also associated with higher mosquito numbers (*χ*^2^ = 11.29, *df* = 4, *P* = 0.024). As above, the application of the Bonferroni-corrected *P*-value threshold would suggest that only the use of fogging can be safely considered associated with lower mosquito numbers.

### Geckos and mosquito abundance

A total of 297 geckos were caught, of which 133 (45%) were in the urban area and 164 (55%) in the rural area. Close to 28% of the geckos were collected in bathrooms (*N* = 82), followed by bedrooms (*N* = 75; 25%), living rooms (*N* = 74; 25%) and kitchens (*N* = 66; 22%). Geckos were caught on the lower (*N* = 102; 34%), intermediate (*N* = 94; 32%) and upper levels (*N* = 101; 34%).

Univariable fitting of the gecko number caught revealed no significant association of gecko number with either the *Ae. aegypti* or *Culex* spp. number caught on sticky traps. Using the total number of mosquitoes caught by aspiration again revealed no association of gecko number with *Ae. aegypti* or *Culex* spp. mosquito number in rural settings. However, there were significant associations of mosquito numbers with gecko numbers in the urban sites. There was a negative association between gecko number and total number of *Ae. aegypti* caught by aspiration (*χ*^2^ = 12.61, *df* = 1, *P* < 0.001). By contrast, there was a positive association between gecko number and the total number of *Culex* spp. caught by aspiration (*χ*^2^ = 7.32, *df* = 1, *P* = 0.008). These associations remained in a multivariable analysis that included the above-identified significant socioenvironmental variables.

## Discussion

This study identified a number of aspects of *Ae. aegypti* mosquito resting behavior and environmental factors therewith associated. We found that the majority of female *Ae. aegypti* mosquitoes rested in bedrooms (35–39% of specimens) and bathrooms (ca. 30%) and at intermediate heights (48–53% of specimens). Similar results were found in Trinidad, Mexico and Panama, where female *Ae. aegypti* generally rested in bedrooms, but less so in bathrooms [7, 8, 10]. In addition, *Culex* spp. were also predominantly found in bedrooms and bathrooms and at low and intermediate heights. The similarities in place and height of resting across the genera are interesting but must be treated with caution, especially as the *Culex* spp. were not identified to the species level. Mosquitoes are abundant in bedrooms likely because people spend a comparatively long time in this room while sleeping, attracting blood-seeking mosquitoes by their body heat and carbon dioxide [20]. Bathrooms in Thailand commonly have open water storage containers for washing and flushing, creating a humid environment that may attract ovipositing mosquitoes [21].

The tendency for resting to predominantly occur at low (< 0.75 m) to intermediate heights (0.75–1.5 m) was observed for both genera. At lower heights there is less air movement, and it is generally darker than the wall near the ceiling and often lit by light bulbs. Furthermore, ceiling fans are often installed, which may interfere with mosquito resting. Finally, at lower heights, there are often hanging objects such as clothes, towels and mosquito nets as well as furniture that create sheltered dark sites, offering an ideal hiding place to rest and digest [22]. Mosquitoes will rest on hanging objects or are attracted to used clothes emitting human odors [23].

For both genera of mosquitoes, there was a tendency for numbers to decrease in the afternoon, suggesting that the mosquitoes were heading outdoors, potentially looking for oviposition sites. Oviposition has been found to peak in late afternoon to early evening [24]. This might explain why the numbers of *Ae. aegypti* were observed to increase in the living room in the afternoons, such rooms being the most juxtaposed to the outdoors. Such exiting behavior is thus likely important to take into account for similar studies.

Several of these explanations as to why the mosquitoes were thus distributed are supported by observed associations with the environmental features of the houses. Notably, hanging clothes at intermediate heights was associated with increased numbers of *Ae. aegypti* in rural settings. The absence of eaves and low wind flow were associated with more *Culex* spp. in urban settings, although the strength of the associations observed was weak. In addition to such house characteristics, it was notable that infrequent use of fogging or larval control was associated with increased numbers of *Culex* spp. and *Ae. aegypti*. Whilst this would make sense, the subjective nature of memory on how frequently vector control was carried out may generate a bias and thus be treated with caution. Identifying household features that provide a conducive environment for mosquitoes can contribute to efforts for implementing push–pull strategies to make houses less attractive [25]. This coupled with knowledge on resting behavior with respect to height and room type can also guide the physical implementation of irritant and spatial repellent insecticides [26]. Whilst the major focus of this work was to identify the optimal sites for use of targeted IRS, clearly clothes and bathrooms are not going to be usefully protected by the IRS. However, spatial repellents using volatile pyrethroids, such as metofluthrin, have recently shown considerable promise in field conditions and have even been found to reduce indoor mosquito densities to levels observed with targeted IRS [27, 28]. Such spatial repellents may well be of significant value in conditions where IRS will not be effective or cannot be implemented.

Comparing the performance of active aspiration versus passive sticky traps to measure mosquito abundance, we found that, broadly, both methods revealed the same resting behavior tendencies, suggesting that low-cost, low-manpower sticky traps offer a viable alternative to aspirators, even if their efficiency (mosquitoes per time) is lower.

Few have studied potential links between mosquito predators, food web interactions and mosquito vector control [29, 30]. Geckos frequently feed on mosquitoes and could thus act as a vector control measure under the right spatial, temporal and optimal forage conditions [16]. However, geckos also prey on common mosquito predators, such as spiders; therefore, geckos might affect mosquito abundance both positively and negatively depending on their foraging preferences [16]. This has led to the proposal that geckos might reduce predator populations and increase the risk of dengue epidemics [31]. Our study identified a significant negative association between geckos caught on the sticky trap and *Ae. aegypti* mosquitoes collected by aspiration in rural settings, indicating a protective effect by the presence of more geckos. On the other hand, *Culex* spp. were positively associated with geckos. This might suggest that the predation by geckos favored *Culex* spp. potentially through the intermediate predation of spiders, whereas *Ae. aegypti* was directly affected by gecko predation. However, these results only applied to the urban settings and require a more detailed study.

### Limitations

A limitation of our study was that we did not distinguish between mosquitoes resting on clothes compared to walls. This was due to methodological complications. However, it would be important for vector control strategies to know whether mosquitoes rest on clothes more than on walls. While it is possible to use IRS on walls, IRS cannot be done if mosquitoes rest on clothes. Furthermore, IRS in bathrooms is not likely to be effective, and other strategies should be employed in such situations. Another limitation of the study was the absence of meteorological data. However, as the villages were sampled sequentially and were taken into account in the analyses, the major findings of the work are unlikely to have been significantly altered by the inclusion of meteorological data. Furthermore, the results were so consistent across the sites whether using aspirators or sticky traps, that although the effect of changes in humidity, rainfall and temperature may have altered total numbers, the behavioral trends remain. Although data were analyzed separately by urban/rural setting, there might be heterogeneity within areas, especially urban areas, for example depending on rich and poor neighborhoods. We did not capture this potential urban heterogeneity. Another limitation is in the sampling strategy used for mechanical aspiration, which always started at the lower wall heights and then progressively moved upwards. Sampling might have been better designed to start at different heights in a randomly selected height protocol. However, that the mosquitoes were predominantly found resting at the lower heights would suggest that any disturbance of mosquitoes pushing them to higher heights did not happen. Finally, the gecko collections were not an initial aim of this study and would therefore benefit from a more targeted study informed by previous studies.

## Conclusion

Our work suggests that vector control using targeted IRS focusing on walls at heights lower than 1.5 m in bedrooms and bathrooms could be part of an integrated effective strategy for dengue vector control. Although we predominantly focused on dengue, *Culex* spp. are vectors of a number of arboviruses in the region, and thus our work provides an evidence base for understanding vector control for such vector-borne pathogens. Finally, this study highlights the importance of regular mosquito control, which can be targeted through education programs.

## Supplementary Information

**Additional file 1: Table S1.** *Ae. aegypti* mosquitoes collected by mechanical battery-driven aspirator differentiated by collection time, room and wall height above the floor in (A) rural areas and (B) urban areas in northeastern Thailand, 2019.

**Additional file 2: Table S2.** *Culex* spp. mosquitoes collected by mechanical battery-driven aspirator differentiated by collection time, room and wall height above floor in (A) rural areas and (B) urban areas in northeastern Thailand, 2019.

**Additional file 3: Table S3.** *Ae. aegypti* mosquitoes collected by sticky traps differentiated by collection room and wall height above floor in (A) rural areas and (B) urban areas in northeastern Thailand, 2019.

**Additional file 4: Table S4.** *Culex* spp. mosquitoes collected by sticky traps differentiated by collection room and wall height above floor in (A) rural areas and (B) urban areas in northeastern Thailand, 2019.

**Additional file 5: Table S5.** Household environmental and socioeconomic characteristics.

**Additional file 6: Table S6.** Univariable *P*-values for association analyses of socioeconomic and environmental variables with *Aedes aegypti* abundance.

**Additional file 7: Table S7.** Univariable *P*-values for association analyses of socioeconomic and environmental variables with *Culex* spp. abundance.

**Additional file 8: Dataset S1.** Dataset on mosquito resting and gecko collections.

**Additional file 9: Dataset S2.** Dataset on socioenvironmental characteristics.

## Data Availability

Relevant available data are found in the Supplementary Information Additional file 8 and Additional file 9.

## Abbreviations

cDNA: Complementary DNA
DENV: Dengue virus
GLMM: Generalized linear mixed model
IRS: Indoor residual spraying
SD: Standard deviation
SEM: Standard error of the mean
