# Supplementary material for: Indoor resting behavior of Aedes aegypti (Diptera: Culicidae) in northeastern Thailand
Source: Parasit Vectors. 2023 Apr 14;16:127. doi: 10.1186/s13071-023-05746-9 (PMC10103527; doi:10.1186/s13071-023-05746-9)
Supplement: Supplementary file 1 — Additional file 1: Table S1. Ae. aegypti mosquitoes collected by mechanical battery-driven aspirator differentiated by collection time, room and wall height above the floor in (A) rural areas and (B) urban areas in northeastern Thailand, 2019. [file 13071_2023_5746_MOESM1_ESM.docx]

**Table S1.** *Ae. aegypti* mosquitoes collected by mechanical battery-driven aspirator differentiated by collection time, room and wall height above floor in A) rural areas and B) urban areas in northeastern Thailand, 2019.

| **A. Rural areas** | | | | | | | | | | | |
| --- | --- | --- | --- | --- | --- | --- | --- | --- | --- | --- | --- |
| **Factors** | **Female** | | |  | **Male** | | |  | **Total** | | |
|  | **No. (%)** | **Range** | **Mean ±SD** |  | **No. (%)** | **Range** | **Mean ±SD** |  | **No. (%)** | **Range** | **Mean ±SD** |
| **Collection time** | | | | | | | | | | | |
| 08:00-12:00 | 317 (60.5) | 0-21 | 0.39 ± 1.37 |  | 249 (57.2) | 0-18 | 0.31 ± 1.05 |  | 566 (59.0) | 0-28 | 0.69 ± 2.10 |
| 13:00-17:00 | 207 (39.5) | 0-8 | 0.33 ± 1.05 |  | 186 (42.8) | 0-12 | 0.30 ± 1.00 |  | 393 (41.1) | 0-13 | 0.63 ± 1.75 |
| **Room** | | | | | | | | | | | |
| Bedroom | 219 (41.8) | 0-21 | 0.61 ± 1.76 |  | 183 (42.1) | 0-18 | 0.51 ± 1.58 |  | 402 (41.9) | 0-28 | 1.12 ± 2.82 |
| Bathroom | 140 (26.7) | 0-10 | 0.39 ± 1.18 |  | 128 (29.4) | 0-7 | 0.36 ± 0.92 |  | 268 (27.9) | 0-17 | 0.74 ± 1.92 |
| Living room | 122 (23.3) | 0-10 | 0.34 ± 1.08 |  | 91 (20.9) | 0-7 | 0.25 ± 0.79 |  | 213 (22.2) | 0-11 | 0.59 ± 1.56 |
| Kitchen | 43 (8.2) | 0-7 | 0.12 ± 0.63 |  | 33 (7.6) | 0-4 | 0.09 ± 0.45 |  | 76 (7.9) | 0-9 | 0.21 ± 0.90 |
| **Height above floor (meters)** | | | | | | | | | | | |
| <0.75 | 162 (30.9) | 0-7 | 0.34 ± 1.05 |  | 116 (26.7) | 0-5 | 0.24 ± 0.68 |  | 278 (29.0) | 0-10 | 0.58 ± 1.42 |
| 0.75-1.5 | 338 (64.5) | 0-21 | 0.70 ± 1.79 |  | 297 (63.8) | 0-18 | 0.62 ± 1.58 |  | 635 (66.2) | 0-28 | 1.32 ± 2.92 |
| >1.5 | 24 (4.6) | 0-5 | 0.05 ± 0.33 |  | 22 (5.1) | 0-2 | 0.05 ± 0.25 |  | 46 (4.8) | 0-5 | 0.10 ± 0.43 |
| **B. Urban areas** | | | | | | | | | | | |
| **Collection time** | | | | | | | | | | | |
| 08:00-12:00 | 100 (54.3) | 0-10 | 0.14 ± 0.81 |  | 86 (59.7) | 0-7 | 0.12 ± 0.63 |  | 186 (56.7) | 0-16 | 0.26 ± 1.36 |
| 13:00-17:00 | 84 (45.7) | 0-5 | 0.12 ± 0.59 |  | 58 (40.3) | 0-5 | 0.08 ± 0.44 |  | 142 (43.3) | 0-10 | 0.20 ± 0.89 |
| **Room** | | | | | | | | | | | |
| Bedroom | 91 (49.5) | 0-10 | 0.25 ± 1.05 |  | 60 (41.7) | 0-7 | 0.17 ± 0.73 |  | 151 (46.0) | 0-16 | 0.42 ± 1.68 |
| Bathroom | 53 (28.8) | 0-10 | 0.15 ± 0.77 |  | 49 (34.0) | 0-6 | 0.14 ± 0.64 |  | 102 (31.1) | 0-16 | 0.28 ± 1.31 |
| Living room | 40 (21.7) | 0-5 | 0.11 ± 0.54 |  | 33 (22.9) | 0-5 | 0.09 ± 0.46 |  | 73 (22.3) | 0-6 | 0.20 ± 0.82 |
| Kitchen | 0 | 0 | 0 |  | 2 (1.4) | 0-1 | 0.01 ± 0.07 |  | 2 (0.6) | 0-1 | 0.01 ± 0.07 |
| **Height above floor (meters)** | | | | | | | | | | | |
| <0.75 | 15 (8.2) | 0-4 | 0.03 ± 0.30 |  | 17 (8.2) | 0-5 | 0.04 ± 0.30 |  | 32 (9.8) | 0-6 | 0.07 ± 0.50 |
| 0.75-1.5 | 167 (90.8) | 0-10 | 0.35 ± 1.16 |  | 126 (90.8) | 0-7 | 0.26 ± 0.87 |  | 293 (89.3) | 0-16 | 0.61 ± 1.87 |
| >1.5 | 2 (1.1) | 0-2 | 0.02 ± 0.09 |  | 1 (1.1) | 0-1 | 0.01 ± 0.05 |  | 3 (0.9) | 0-2 | 0.02 ± 0.10 |
