# Supplementary material for: Indoor resting behavior of Aedes aegypti (Diptera: Culicidae) in northeastern Thailand
Source: Parasit Vectors. 2023 Apr 14;16:127. doi: 10.1186/s13071-023-05746-9 (PMC10103527; doi:10.1186/s13071-023-05746-9)
Supplement: Supplementary file 2 — Additional file 2: Table S2. Culex spp. mosquitoes collected by mechanical battery-driven aspirator differentiated by collection time, room and wall height above floor in (A) rural areas and (B) urban areas in northeastern Thailand, 2019. [file 13071_2023_5746_MOESM2_ESM.docx]

**Table S2.** *Culex* spp. mosquitoes collected by mechanical battery-driven aspirator differentiated by collection time, room and wall height above floor in A) rural areas and B) urban areas in northeastern Thailand, 2019.

| **A. Rural areas** | | | | | | | | | | | |
| --- | --- | --- | --- | --- | --- | --- | --- | --- | --- | --- | --- |
| **Factors** | **Female** | | |  | **Male** | | |  | **Total** | | |
|  | **No. (%)** | **Range** | **Mean ±SD** |  | **No. (%)** | **Range** | **Mean ±SD** |  | **No. (%)** | **Range** | **Mean ±SD** |
| **Collection time** | | | | | | | | | | | |
| 08:00-12:00 | 408 (70.6) | 0-27 | 0.5 ± 1.80 |  | 218 (52.7) | 0-9 | 0.27 ± 0.84 |  | 626 (63.1) | 0-27 | 0.77 ± 1.98 |
| 13:00-17:00 | 170 29.4) | 0-23 | 0.27 ± 1.33 |  | 196 (47.3) | 0-12 | 0.31 ± 1.27 |  | 366 (36.9) | 0-23 | 0.59 ± 1.83 |
| **Room** | | | | | | | | | | | |
| Bedroom | 266 (46.0) | 0-27 | 0.74 ± 2.64 |  | 60 (14.5) | 0-8 | 0.17 ± 0.68 |  | 326 (32.9) | 0-27 | 0.91 ± 2.74 |
| Bathroom | 168 (29.1) | 0-11 | 0.47 ± 1.39 |  | 135 (32.6) | 0-7 | 0.38 ± 1.16 |  | 303 (30.5) | 0-13 | 0.84 ± 1.84 |
| Living room | 93 (16.1) | 0-9 | 0.26 ± 0.97 |  | 80 (19.3) | 0-9 | 0.22 ± 0.83 |  | 173 (17.4) | 0-9 | 0.48 ± 1.24 |
| Kitchen | 51 (8.8) | 0-6 | 0.14 ± 0.62 |  | 139 (33.6) | 0-12 | 0.39 ± 1.37 |  | 190 (19.2) | 0-12 | 0.53 ± 1.49 |
| **Height above floor (m)** | | | | | | | | | | | |
| <0.75 | 239 (41.3) | 0-27 | 0.50 ± 2.23 |  | 162 (39.1) | 0-12 | 0.34 ± 1.21 |  | 401 (40.4) | 0-27 | 0.84 ± 2.51 |
| 0.75-1.5 | 279 (48.3) | 0-9 | 0.58 ± 1.51 |  | 136 (32.9) | 0-9 | 0.28 ± 1.01 |  | 415 (41.8) | 0-9 | 0.86 ± 1.80 |
| >1.5 | 60 (10.4) | 0-11 | 0.13 ± 0.69 |  | 116 (28.0) | 0-9 | 0.24 ± 0.9 |  | 176 (17.8) | 0-13 | 0.37 ± 1.16 |
| **B. Urban areas** | | | | | | | | | | | |
| **Collection time** | | | | | | | | | | | |
| 08:00-12:00 | 169 (76.5) | 0-12 | 0.23 ± 1.04 |  | 252 (64.0) | 0-19 | 0.35 ± 1.33 |  | 421 (68.5) | 0-19 | 0.58 ± 1.68 |
| 13:00-17:00 | 52 (23.5) | 0-4 | 0.07 ± 0.42 |  | 142 (36.0) | 0-8 | 0.20 ± 0.91 |  | 194 (31.5) | 0-8 | 0.27 ± 1.01 |
| **Room** | | | | | | | | | | | |
| Bedroom | 108 (48.9) | 0-12 | 0.30 ± 1.28 |  | 103 (26.15) | 0-9 | 0.29 ± 1.14 |  | 211 (34.3) | 0-12 | 0.59 ± 1.68 |
| Bathroom | 59 (26.7) | 0-8 | 0.16 ± 0.68 |  | 103 (26.15) | 0-19 | 0.29 ± 1.39 |  | 162 (26.3) | 0-19 | 0.45 ± 1.55 |
| Living room | 37 (16.7) | 0-3 | 0.10 ± 0.50 |  | 62 (15.7) | 0-7 | 0.17 ± 0.75 |  | 99 (16.1) | 0-8 | 0.28 ± 0.97 |
| Kitchen | 17 (7.7) | 0-6 | 0.05 ± 0.39 |  | 126 (32.0) | 0-8 | 0.35 ± 1.18 |  | 143 (23.3) | 0-8 | 0.40 ± 1.26 |
| **Height above floor (m)** | | | | | | | | | | | |
| <0.75 | 79 (35.7) | 0-12 | 0.16 ± 0.84 |  | 177 (44.9) | 0-19 | 0.37 ± 1.40 |  | 256 (41.6) | 0-19 | 0.53 ± 1.65 |
| 0.75-1.5 | 108 (48.9) | 0-8 | 0.23 ± 0.89 |  | 143 (36.3) | 0-8 | 0.30 ± 1.16 |  | 251 (40.8) | 0-8 | 0.52 ± 1.45 |
| >1.5 | 34 (15.4) | 0-12 | 0.07 ± 0.63 |  | 74 (18.8) | 0-9 | 0.15 ± 0.76 |  | 108 (17.6) | 0-12 | 0.23 ± 0.99 |
