# Supplementary material for: Indoor resting behavior of Aedes aegypti (Diptera: Culicidae) in northeastern Thailand
Source: Parasit Vectors. 2023 Apr 14;16:127. doi: 10.1186/s13071-023-05746-9 (PMC10103527; doi:10.1186/s13071-023-05746-9)
Supplement: Supplementary file 3 — Additional file 3: Table S3. Ae. aegypti mosquitoes collected by sticky traps differentiated by collection room and wall height above floor in (A) rural areas and (B) urban areas in northeastern Thailand, 2019. [file 13071_2023_5746_MOESM3_ESM.docx]

**Table S3.** *Ae. aegypti* mosquitoes collected by sticky traps differentiated by collection room and wall height above floor in A) rural areas and B) urban areas in northeastern Thailand, 2019.

| **A. Rural areas** | | | | | | | | | | | |
| --- | --- | --- | --- | --- | --- | --- | --- | --- | --- | --- | --- |
| **Factors** | **Female** | | |  | **Male** | | |  | **Total** | | |
|  | **No. (%)** | **Range** | **Mean ±SD** |  | **No. (%)** | **Range** | **Mean ±SD** |  | **No. (%)** | **Range** | **Mean ±SD** |
| **Room** | | | | | | | | | | | |
| Bedroom | 131 (39.2) | 0-9 | 4.37 ± 2.43 |  | 118 (42.1) | 0-8 | 3.93 ± 2.16 |  | 249 (40.6) | 0-17 | 8.30 ± 4.24 |
| Bathroom | 98 (29.3) | 0-7 | 3.27 ± 2.27 |  | 84 (30.0) | 0-8 | 2.80 ± 2.23 |  | 182 (29.6) | 0-15 | 6.07 ± 4.07 |
| Living room | 67 (20.1) | 0-3 | 2.23 ± 1.43 |  | 51 (18.2) | 0-5 | 1.70 ± 1.58 |  | 118 (19.2) | 0-9 | 3.93 ± 2.64 |
| Kitchen | 38 (11.4) | 0-5 | 1.27 ± 1.01 |  | 27 (9.6) | 0-2 | 0.90 ± 0.80 |  | 65 (9.6) | 0-5 | 2.17 ± 1.46 |
| **Height above floor (m)** | | | | | | | | | | | |
| <0.75 | 102 (30.5) | 0-7 | 2.55 ± 1.60 |  | 78 (27.9) | 0-6 | 1.95 ± 1.93 |  | 180 (29.3) | 0-12 | 4.50 ± 3.06 |
| 0.75-1.5 | 178 (53.3) | 0-9 | 4.45 ± 2.25 |  | 154 (55.0) | 0-8 | 3.85 ± 2.23 |  | 332 (54.1) | 0-17 | 8.30 ± 4.21 |
| >1.5 | 54 (16.2) | 0-7 | 1.35 ± 1.41 |  | 48 (17.1) | 0-4 | 1.20 ± 1.02 |  | 102 (16.6) | 0-9 | 2.55 ± 2.00 |
| **B. Urban areas** | | | | | | | | | | | |
| **Room** | | | | | | | | | | | |
| Bedroom | 66 (35.3) | 0-6 | 2.20 ± 1.52 |  | 56 (36.6) | 0-7 | 1.87 ± 1.70 |  | 122 (35.9) | 0-10 | 4.07 ± 2.69 |
| Bathroom | 59 (31.6) | 0-4 | 1.97 ± 1.13 |  | 49 (32.0) | 0-4 | 1.63 ± 1.22 |  | 108 (31.8) | 0-7 | 3.60 ± 2.06 |
| Living room | 37 (19.8) | 0-4 | 1.23 ± 1.14 |  | 30 (19.6) | 0-3 | 1.00 ± 0.98 |  | 67 (19.7) | 0-6 | 2.23 ± 1.76 |
| Kitchen | 25 (13.4) | 0-4 | 0.83 ± 0.95 |  | 18 (11.8) | 0-3 | 0.60 ± 0.72 |  | 43 (12.6) | 0-7 | 1.43 ± 1.52 |
| **Height above floor (m)** | | | | | | | | | | | |
| <0.75 | 65 (34.8) | 0-4 | 1.63 ± 1.17 |  | 51 (33.3) | 0-7 | 1.28 ± 2.01 |  | 116 (34.1) | 0-10 | 2.90 ± 2.01 |
| 0.75-1.5 | 89 (47.6) | 0-6 | 2.23 ± 1.40 |  | 77 (50.3) | 0-6 | 1.93 ± 2.44 |  | 166 (48.8) | 0-9 | 4.15 ± 2.44 |
| >1.5 | 33 (17.6) | 0-4 | 0.83 ± 0.93 |  | 25 (16.3) | 0-3 | 0.63 ± 1.48 |  | 58 (17.1) | 0-5 | 1.45 ± 1.48 |
