# Supplementary material for: Indoor resting behavior of Aedes aegypti (Diptera: Culicidae) in northeastern Thailand
Source: Parasit Vectors. 2023 Apr 14;16:127. doi: 10.1186/s13071-023-05746-9 (PMC10103527; doi:10.1186/s13071-023-05746-9)
Supplement: Supplementary file 4 — Additional file 4: Table S4. Culex spp. mosquitoes collected by sticky traps differentiated by collection room and wall height above floor in (A) rural areas and (B) urban areas in northeastern Thailand, 2019. [file 13071_2023_5746_MOESM4_ESM.docx]

**Table S4.** *Culex* spp*.* mosquitoes collected by sticky traps differentiated by collection room and wall height above floor in A) rural areas and B) urban areas in northeastern Thailand, 2019.

| **A. Rural areas** | | | | | | | | | | | |  |
| --- | --- | --- | --- | --- | --- | --- | --- | --- | --- | --- | --- | --- |
| **Factors** | **Female** | | |  | **Male** | | |  | **Total** | | |  |
|  | **No. (%)** | **Range** | **Mean ±SD** |  | **No. (%)** | **Range** | **Mean ±SD** |  | **No. (%)** | **Range** | **Mean ±SD** |  |
| **Room** | | | | | | | | | | | |  |
| Bedroom | 60 (20.5) | 0-8 | 2.00 ± 2.12 |  | 57 (21.8) | 0-9 | 1.90 ± 2.40 |  | 117 (21.1) | 0-12 | 3.90 ± 3.07 |  |
| Bathroom | 74 (25.3) | 0-8 | 2.47 ± 2.33 |  | 74 (28.2) | 0-11 | 2.47 ± 2.56 |  | 148 (26.7) | 0-13 | 4.93 ± 3.32 |  |
| Living room | 112 (38.2) | 0-12 | 3.73 ± 2.77 |  | 65 (24.8) | 0-8 | 2.17 ± 2.04 |  | 177 (31.9) | 0-14 | 5.90 ± 3.40 |  |
| Kitchen | 47 (16.0) | 0-5 | 1.57 ± 1.45 |  | 66 (25.2) | 0-6 | 2.20 ± 1.85 |  | 113 (20.3) | 0-11 | 3.77 ± 2.74 |  |
| **Height above floor (m)** | | | | | | | | | | | |  |
| <0.75 | 103 (35.2) | 0-12 | 2.58 ± 2.52 |  | 71 (27.1) | 0-6 | 1.78 ± 1.61 |  | 174 (31.4) | 0-14 | 4.35 ± 3.17 |  |
| 0.75-1.5 | 111 (37.9) | 0-8 | 2.78 ± 2.45 |  | 105 (40.1) | 0-9 | 2.63 ± 2.60 |  | 216 (38.9) | 0-13 | 5.40 ± 3.47 |  |
| >1.5 | 79 (26.9) | 0-8 | 1.98 ± 1.98 |  | 86 (32.8) | 0-11 | 2.15 ± 2.27 |  | 165 (29.7) | 0-13 | 4.13 ± 2.93 |  |
| **B. Urban areas** | | | | | | | | | | | |  |
| **Room** | | | | | | | | | | | |  |
| Bedroom | 50 (27.9) | 0-7 | 1.67 ± 1.97 |  | 60 (31.2) | 0-9 | 2.00 ± 2.29 |  | 110 (29.6) | 0-12 | 3.67 ± 2.95 |  |
| Bathroom | 33 (18.4) | 0-5 | 1.10 ± 1.24 |  | 48 (25.0) | 0-4 | 1.60 ± 1.48 |  | 81 (21.8) | 0-7 | 2.70 ± 2.12 |  |
| Living room | 55 (30.7) | 0-7 | 1.83 ± 1.64 |  | 38 (19.8) | 0-4 | 1.27 ± 1.39 |  | 93 (25.1) | 0-7 | 3.10 ± 2.09 |  |
| Kitchen | 41 (22.9) | 0-8 | 1.37 ± 1.75 |  | 46 (24.0) | 0-5 | 1.53 ± 1.53 |  | 110 (29.6) | 0-12 | 3.67 ± 2.95 |  |
| **Height above floor (m)** | | | | | | | | | | | |  |
| <0.75 | 48 (26.8) | 0-5 | 1.20 ± 1.44 |  | 89 (46.4) | 0-9 | 2.23 ± 1.97 |  | 137 (36.9) | 0-12 | 3.43 ± 2.54 | |
| 0.75-1.5 | 58 (32.4) | 0-6 | 1.45 ± 1.47 |  | 53 (27.6) | 0-5 | 1.33 ± 1.40 |  | 111 (29.9) | 0-8 | 2.78 ± 2.21 | |
| >1.5 | 73 (40.8) | 0-8 | 1.83 ± 2.04 |  | 50 (26.0) | 0-6 | 1.25 ± 1.56 |  | 123 (33.2) | 0-8 | 3.08 ± 2.21 | |
